# Threshold effects of physical activity and cognitive function among older adults with diabetes mellitus in NHANES 2011–2014

**DOI:** 10.1007/s40520-025-03255-6

**Published:** 2025-12-29

**Authors:** Jigang Ren, Huibiao Li, Li Liufu, YuPing Yang, Danting Long, Hong Liu, Lidian Chen

**Affiliations:** 1 College of Rehabilitation Medicine, Fujian University of Traditional Chinese Medicine, Fuzhou, China; 2 Department of Traditional Chinese Medicine, Affiliated Hospital of North Sichuan Medical College, Nanchong, Sichuan China; 3 Key Laboratory of Orthopedics and Traumatology of Traditional Chinese Medicine and Rehabilitation, Ministry of Education, Fujian University of Traditional Chinese Medicine, Fuzhou, China; 4 Fujian University of Traditional Chinese Medicine, No.1 Qiuyang Road, Minhou Shangjie, Fuzhou, 350000 China

**Keywords:** Diabetes mellitus, Cognitive function, Physical activity, Older adults, Threshold effect

## Abstract

**Background:**

Deterioration of cognitive function with aging is a significant public health issue, particularly in individuals with diabetes mellitus (DM). Exercise has been shown to enhance cognitive function. However, the threshold effect of physical activity on cognitive function in older adult people with DM remains unclear.

**Methods:**

This study analyzed data from 925 older participants (aged 60 and above) derived from the National Health and Nutrition Examination Survey (NHANES) conducted between 2011 and 2014, representing a total weighted respondent count of 13,824,651. Cognitive function was evaluated with the Animal Fluency test (AFT) and Digit Symbol Substitution test (DSST). To assess the relationship between physical activity and cognitive function, we applied weighted linear regression models coupled with restricted cubic spline analysis. Furthermore, a two-piecewise linear regression model was utilized to detect any potential threshold effect of exercise on cognitive function.

**Results:**

The results indicated a positive correlation between physical activity and cognitive function scores on the AFT and DSST after adjusting for potential confounders. Threshold analyses showed a consistent positive relationship for AFT scores at less than 490 MET-min/week of physical activity [β (95% CI) = 0.45 (0.20, 0.70), *p* = 0.001] and for DSST scores at less than 1,120 MET-min/week [β (95% CI) = 0.55 (0.20, 0.89), *p* = 0.004]. However, when the exercise volume reached these two inflection points, a saturation effect occurred.

**Conclusion:**

This study shows a clear inverted U-shaped relationship between physical activity and cognitive function in older adults with DM. Cognitive benefits do not increase with higher exercise volume and approximately 490 MET-minutes/week appears to be the optimal dose for preserving cognitive function in this population. Additional research is necessary to confirm these findings in future studies using objective, precise measures such as pedometers and accelerometers.

**Supplementary Information:**

The online version contains supplementary material available at 10.1007/s40520-025-03255-6.

## Introduction

As the global population continues to age, the deterioration of cognitive function, exemplified by progressive impairments in memory, judgment, language, and attention, has become an increasingly pressing public health concern [1, 2]. Cognitive decline, particularly when it is persistent and progressive, may indicate an increased risk of mild cognitive impairment (MCI), which can further increase the likelihood of developing dementia [3]. A meta-analysis of 51 studies revealed that the current global prevalence of MCI is 23.7% in geriatric population [4]. Additionally, it is estimated that in 2019, approximately 55.2 million people worldwide were affected by dementia, a number projected to escalate to 152.8 million by 2050 [5, 6]. Cognitive impairments and their associated conditions not only diminish quality of life and overall functional capacity in older adults but also impose significant burdens on their families and society [7–9].

In modern society, diabetes mellitus (DM) has become increasingly prevalent, affecting approximately 589 million adults worldwide [10]. Prevalence exceeds 29% among those aged 65 and older [11, 12]. Previous studies have identified DM as a significant risk factor for cognitive decline, which may lead to and exacerbate cognitive impairment and dementia [13–16]. Recent findings suggest that 25–36% of individuals with DM may develop MCI, and they face nearly double the risk of dementia compared to those without DM [17, 18]. Meanwhile cognitive impairment manifested as reduced memory comprehension and executive function diminishes patients capacity for blood glucose self-management. This includes diabetes knowledge, insulin injection and dose adjustment, and accuracy of blood glucose records, thereby worsening DM and accelerating further cognitive decline [19]. Current diabetes prevention policies focus almost exclusively on glycaemic and cardiovascular targets, leaving the reciprocal pathway between DM and cognitive dysfunction unaddressed [20]. Randomised trials and meta-analyses show that structured physical activity improves glycaemic control and concurrently reduces cognitive decline in older adults with type 2 diabetes [21–23]. Therefore, integrated interventions that embed individualised physical activity prescriptions within combined glycaemic and cognitive lifestyle programmes are urgently needed to protect cognitive function while curbing diabetes progression in this population.

Physical activity (PA) and regular exercise, as a lifestyle with several health benefits, have been identified as an effective strategy to mitigate cognitive decline in the older adults [24, 25]. PA is also recognised as a key element in preventing and managing chronic diseases, particularly DM, and has become an integral component of therapeutic strategies [26, 27]. Extensive research has confirmed that exercise can significantly reduce the risk of developing type 2 diabetes and improve glycemic management and overall health in those with diabetes [28, 29]. Meanwhile, moderate exercise up-regulates brain derived neurotrophic factor (BDNF) levels and restores insulin sensitivity as well as brain insulin signaling, thereby slowing diabetes-related cognitive decline [30, 31]. Therefore, regular exercise is a simple yet effective strategy to reduce the prevalence of diabetes mellitus, maintain cognitive health, and enhance overall function among older adults.

Although exercise is well established for protecting cognitive function, large population studies focusing on older adults with DM remain limited. It is also unclear whether a threshold effect exists in this population, where exercise benefits might plateau or diminish [32, 33]. In this study, using data from the National Health and Nutrition Examination Survey, we aimed to investigate the relationship between PA and cognitive function in older adults with DM and to evaluate the threshold effects of exercise on cognitive function in this population. The findings could provide evidence-based guidance for lifestyle recommendations targeting cognitive preservation in older adults with DM.

## Methods

### Study design and population

Study data were from the 2011–2014 cycle of the National Health and Nutrition Examination Survey (NHANES), a comprehensive, population-based survey conducted by the National Center for Health Statistics (NCHS) to assess civilians’ health and nutritional conditions [34]. NHANES employed a sophisticated, multistage probability sampling design to encompass individual representative of the general, non-institutionalized population across all age groups. The survey is composed of a structured interview conducted in the home environment for provided information about demographics, socioeconomic status, and health status, trailed by a standardized health examination consisting of a physical examination and laboratory tests. The survey protocol was approved by the Institutional Review Board of the NCHS. All participants or proxies had given written informed consent.

**Fig. 1:**
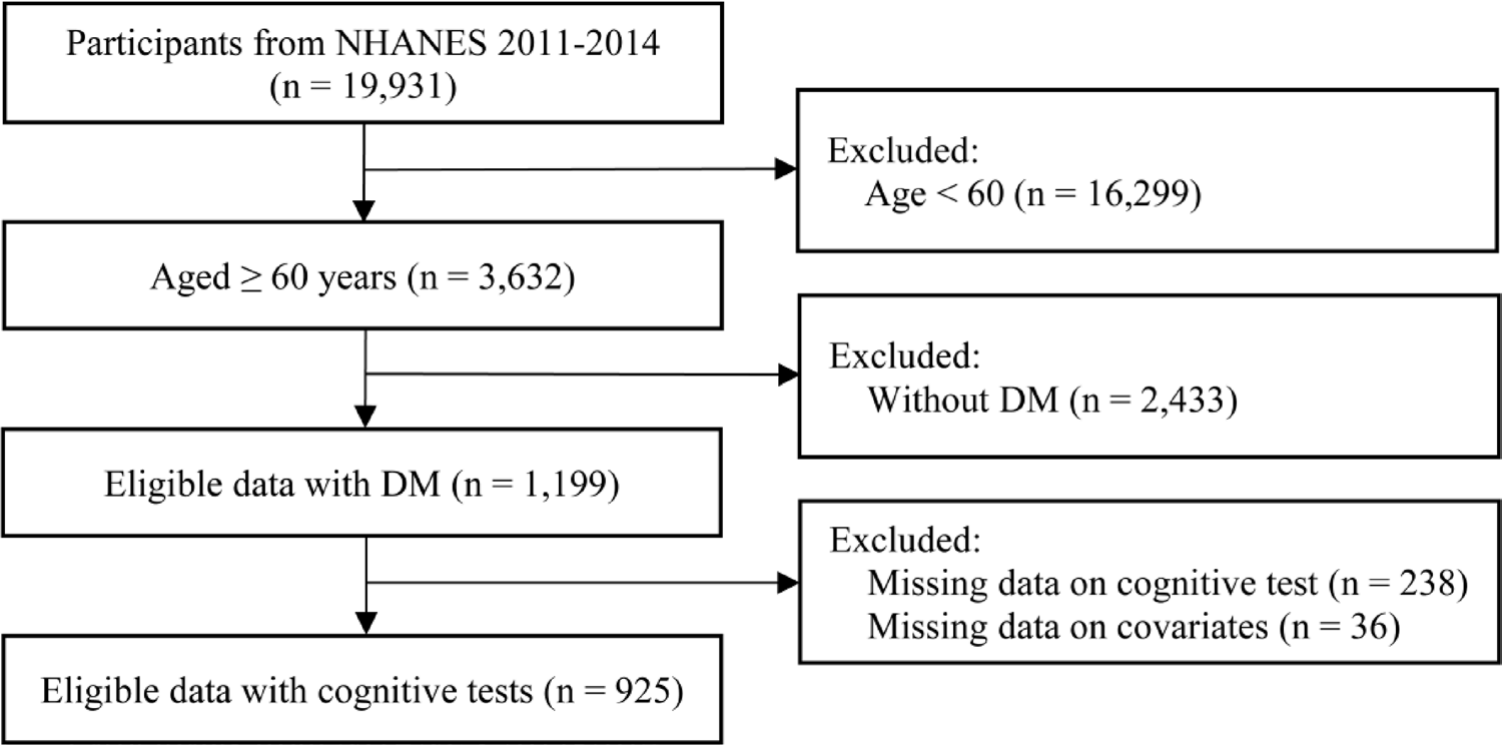
Flowchart of participants selection from NHANES 2011–2014. NHANES: National Health and Nutrition Examination Survey; DM: Diabetes Mellitus

Initially, 19,931 participants were enrolled, with 3,632 individuals aged ≥ 60 years. After excluding those with incomplete cognitive or covariate data, 925 samples were enrolled in this study for final analysis (Fig. 1).

## Exposure measurement

The diagnosis of DM was based on the American Diabetes Association criteria [35, 36]. Participants were considered to have DM if they met any one of the following criteria: (1) self-reported previous diagnosis of DM, (2) hemoglobin A1c (HbA1c) ≥ 6.5%, (3) fasting plasma glucose (PG) ≥ 7.0 mmol/L, (4) two-hour PG during oral glucose tolerance test (OGTT) ≥ 11.1 mmol/L, (5) random PG ≥ 11.1 mmol/L, and (6) using anti-diabetic medication or insulin. Data on PA were collected during home interviews using the physical activity questionnaire. PA was defined as sports, fitness, and other recreational physical activities during leisure time, excluding work-related physical activities. To calculate the weekly metabolic equivalent of task (MET) minutes for each participant, we used moderate and vigorous PA (MVPA) measures [37, 38]. The MVPA considered that two minutes of moderate PA were equivalent to one minute of vigorous PA. Subsequently, the standard MET value of each activity was multiplied by the weekly minutes of MVPA to calculate the MET-minutes. PA intensity was classified as moderate (4 MET) or vigorous (8 MET) [39]. In this study, to account for the cumulative effects of PA, the volume was measured in units of 100 MET-min/week. Based on the World Health Organization (WHO) guidelines [40, 41] and previous studies [42], the volume of PA was categorized into three distinct levels: none (< 1 MET-min/week), inadequate activity (1 to 600 MET-min/week), and adequate activity (≥ 600 MET-min/week) for analysis.

## Cognitive function modules

In NHANES, cognitive function in participants aged 60 and older was assessed using Animal Fluency test (AFT) and Digit Symbol Substitution test (DSST). These assessments were administered by trained interviewers during household interviews or at Mobile Examination Centers (MEC). The AFT is a verbal category fluency test that evaluates executive function, semantic memory, and processing speed by requiring participants to list as many animals as possible within 1 min [43]. The DSST, a cognitive performance test from the Wechsler Adult Intelligence Scale-III, comprehensively measures cognitive functioning, including processing speed, visual scanning, sustained attention, and short-term memory [44]. This test involved a paper-based number-symbol matching task. A key at the top linked nine numbers to unique symbols, and participants had 2 min to copy the symbols into 133 adjacent boxes. The maximum score possible was 40 for AFT and 100 for DSST respectively. In every test, a higher score indicated superior cognitive function. Detailed information on the paradigm of the two cognitive tests was described on the NHANES website: http://www.cdc.gov/nchs/nhanes/.

## Covariates

Following previous research [45, 46], demographic characteristics, including age, gender (male, female), race (Mexican American, Non-Hispanic White, Non-Hispanic Black, other race), marital status (married or living with partner, widowed/divorced/separated/never married), education level (below high school, high school, college or above), and family poverty income ratio (PIR) [low income (< 1), middle income (≥ 1 and < 3), and high income (≥ 3)], was extracted from the demographic questionnaire. Additionally, alcohol intake status and smoking status were assessed through separate questionnaires. Alcohol intake status was classified as “yes” or “no” (in the past 12 months had at least 12 drinks of any kind of alcoholic beverage were characterized as drinker), based on questionnaire responses. Smoking status was categorized as never smoked (smoked < 100 cigarettes), former smoker (not currently smoking but smoked ≥ 100 cigarettes), and current smoker (≥ 100 cigarettes and currently smoking every day or on some days). Furthermore, we evaluated the individuals’ body mass index (BMI) (under & healthy weight < 25 kg/m^2^, overweight ≥ 25 to 30 kg/m^2^, obesity ≥ 30 kg/m^2^). Finally, participants’ disease histories were evaluated. Participants were deemed to have hypertension if they met the following criteria: self-reported diagnosis of hypertension or taking medication for hypertension, or mean systolic blood pressure ≥ 140mmHg, or mean diastolic blood pressure ≥ 90mmHg. The other medical conditions were defined as hypertension or self-reported angina, coronary heart disease, heart attack, congestive heart failure, or stroke.

### Statistical analysis

All analyses were conducted following the recommended NHANES analysis guidelines and applying the appropriate sample weights to each participant. Survey weights from MEC interviews spanning four years of NHANES data (2011–2014) were applied to address non-response, non-coverage, and varying probabilities of selection. Continuous variables are expressed as weighted means with standard error (SE), while categorical variables are reported as numbers and weighted proportions.

In this study, three model analyses were performed: a crude model unadjusted for covariates, Model 1 adjusted for age, gender, and race, and Model 2 further adjusted for marital status, education, PIR, alcohol consumption, smoking, BMI, and other medical conditions. A weighted linear regression model was used to examine the relationship between PA and cognitive function test outcomes. To explore the threshold effect and adjust for all potential confounders, a two-segment linear regression model was constructed. The threshold of PA (100 * MET-minutes/week) was identified using a recurrence method. This method involves identifying the inflection point within a predefined range and selecting the most probable one. The log-likelihood ratio test compared the two-segment model with a single-line model, while restricted cubic spline (RCS) analysis with three optimal knots assessed nonlinearity. Furthermore, we performed stratified analyses to assess the influence of various covariates on the relationship between PA and cognitive function. All analyses used statistical software (R version 4.4.1) with significance established at a *p*-value of 0.05 or less.

## Results

### Baseline characteristics

In the final analysis, 925 participants aged 60 or older were included, and represented for a weighted population of 13,824,651. Table 1 displays the participants’ demographic information. Study participants were an average of 69.54 years old, and consisted of nearly equal numbers of men and women. Moreover, majority of participants were Non-Hispanic Whites (70.2%), had higher education levels (76.8%), had obesity (55.8%), did not exercise regularly (65.3%), and had other medical conditions (84.9%). The mean physical activity of all participants was 357 MET-minutes/week. In addition, the average score of the two tests related to cognitive function (AFT, and DDST) were 17.09, and 46.17, respectively.

**Table 1:** Weighted demographic characteristics of participants in NHANES 2011–2014

Characteristics	Overall (*N* = 925)
Age, year, N (%)	
[60, 70)	496 (52.4)
[70, 80]	429 (47.6)
Gender, N (%)	
Male	483 (50.2)
Female	442 (49.8)
Race, N (%)	
Mexican American	102 (5.4)
Non-Hispanic White	356 (70.2)
Non-Hispanic Black	266 (12.3)
Other Race	201 (12.1)
Marital status, N (%)	
Married/Living with partner	526 (62.2)
Widowed/Divorced/Separated/Never married	399 (37.8)
Education, N (%)	
Below High school	138 (8.8)
High school	165 (14.4)
College or above	622 (76.8)
PIR, N (%)	
< 1	257 (18.6)
[1, 3)	404 (42.6)
≥ 3	264 (38.8)
Alcohol drinkers, N (%)	
No	322 (32.8)
Yes	603 (67.2)
Smokers, N (%)	
Never	425 (44.9)
Former	382 (44.3)
Current	118 (10.8)
BMI, N (%)	
< 25	155 (14.8)
[25, 30)	293 (29.4)
≥ 30	477 (55.8)
Other medical conditions, N (%)	
No	145 (15.1)
Yes	780 (84.9)
PA (100 * MET-minutes/week, continuous), mean ± SE	3.57 (0.45)
PA (category), N (%)	
None	597 (65.3)
Inadequate activity	135 (14.5)
Adequate activity	193 (20.2)
Score of the AFT, mean ± SE	17.09 (0.25)
Score of the DSST, mean ± SE	46.17 (0.91)

NHANES: National Health and Nutrition Examination Survey; PIR: Poverty Income Ratio; BMI: Body Mass Index; PA: Physical Activity; MET: Metabolic Equivalent of Task; AFT: the Animal Fluency test; DSST: the Digit Symbol Substitution test; SE: Standard Error

### The association between PA and cognitive function in older adults with DM

Table 2 presents the results of weighted linear regression analyses examining the association between PA and cognitive function test performance, as measured by the AFT and DSST. For the AFT, higher exercise volume was consistently associated with better cognitive performance when PA was analyzed as a continuous variable. This association remained statistically significant across all regression models, even after sequential adjustment for covariates [Crude Model, β (95% CI) = 0.10 (0.06, 0.14), *p* < 0.001; Model 1, β (95% CI) = 0.09 (0.05, 0.13), *p* < 0.001; Model 2, β (95% CI) = 0.09 (0.05, 0.13), *p* < 0.001]. Furthermore, this association persisted when exercise was evaluated as a categorical variable. From the Crude Model to Model 2, with all covariates included, sufficient exercise volume (more than 600 MET-minutes/week) maintained a positive association with AFT scores [β (95% CI) = 2.24 (1.28, 3.20), *p* < 0.001]. Moreover, the stratified analysis detailed in supplementary Fig. S1 and Fig. S2 corroborated the consistency and robustness of these associations across different subgroups.

In contrast, for the DSST, exercise volume showed no statistically significant association with cognitive performance when analyzed as a continuous variable, even after full adjustment for covariates [Model 2: β (95% CI) = 0.14 (-0.06, 0.34), *p* = 0.164]. However, when exercise volume was evaluated as a categorical variable, adequate exercise (more than 600 MET-minutes/week) demonstrated a significant positive correlation with DSST scores. This association was observed in the Crude Model [β (95% CI) = 6.17 (1.82, 10.51), *p* = 0.007] and remained statistically significant after sequential adjustment for all covariates [β (95% CI) = 4.71 (1.58, 7.84), *p* = 0.006]. As shown in supplementary Fig. S3 and Fig. S4, further stratified analyses across subgroups with diverse demographic profiles demonstrated the consistency of these associations.

**Table 2:** Associations between PA and cognitive function among the older adults with DM in a multiple regression model

		Crude Model		Model 1		Model 2	
		β (95% CI)	*P* - value	β (95% CI)	*P* - value	β (95% CI)	*P* - value
**Score of the AFT**	**PA (as continuous variable)**						
	PA (100*MET-minutes/week)	0.10 (0.06, 0.14)	< 0.001	0.09 (0.05, 0.13)	< 0.001	0.09 (0.05, 0.13)	< 0.001
	**PA (as category variable)**						
	None	Reference		Reference		Reference	
	Inadequate activity	1.90 (0.44, 3.37)	0.013	1.91 (0.45, 3.38)	0.013	1.53 (0.01, 3.06)	0.049
	Adequate activity	2.40 (1.32, 3.47)	< 0.001	2.41 (1.42, 3.41)	< 0.001	2.24 (1.28, 3.20)	< 0.001
**Score of the DSST**	**PA (as continuous variable)**						
	PA (100*MET-minutes/week)	0.18 (-0.03, 0.39)	0.092	0.17 (-0.09, 0.44)	0.189	0.14 (− 0.06, 0.34)	0.164
	**PA (as category variable)**						
	None	Reference		Reference		Reference	
	Inadequate activity	5.81 (1.71, 9.92)	0.007	5.93 (2.40, 9.46)	0.020	2.96 (− 0.79, 6.71)	0.113
	Adequate activity	6.17 (1.82, 10.51)	0.007	6.53 (2.68, 10.38)	0.018	4.71 (1.58, 7.84)	0.006

CI: Confidence Interval; PA: Physical Activity; MET: Metabolic Equivalent of Task; AFT: the Animal Fluency test; DSST: the Digit Symbol Substitution test

Crude Model: no covariates were adjusted

Model 1: age, gender, and race were adjusted

Model 2: age, gender, race, marital status, education, PIR, alcohol drinkers, smokers, BMI, and other medical conditions were adjusted

### Threshold effect analysis

To explore potential threshold effects, we conducted a log-likelihood ratio test (LLR) comparing single-line and segmented regression models, revealing a significant threshold (LLR < 0.01). For the AFT (Table 3), a two-piecewise regression linear model identified an inflection point at 490 MET-minutes/week. To the left of the inflection point, a significant positive link between PA volume and cognitive scores emerged [β (95% CI) = 0.45 (0.20, 0.70), *p* = 0.001]. However, on the right side of this point, this association became nonsignificant [β (95% CI) = 0.01 (-0.07, 0.09), *p* = 0.864], suggesting a saturation effect. Similar findings appeared in the DSST (Table 4). Cognitive scores correlated significantly with PA volume up to 1,120 MET-min/week [β (95% CI) = 0.55 (0.20, 0.89), *p* = 0.004]. Beyond this point, the relationship between PA and cognitive performance lost significance [β (95% CI) = -0.15 (-0.35, 0.05), *p* = 0.121], with an LLR of < 0.001.

**Fig. 2:**
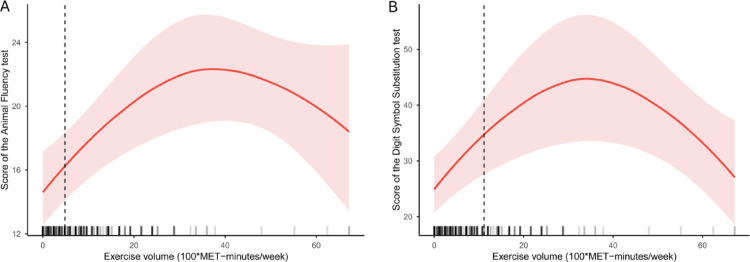
Inverted U-shaped relationship between PA volume with the score of AFT (**A**) and DSST (**B**) in older adults with DM

In Fig. 2, we employed restricted cubic splines to model and visualize the relationship between PA volume and cognitive performance in older adults with DM. The analysis revealed that the effects of exercise on the AFT and DSST exhibited saturation points at 4.9 and 11.2 (100*MET-minutes/week), respectively. Beyond these points, while cognitive performance continued to improve with increased PA volume, the rate of enhancement began to slow down.

**Table 3:** Threshold effect analysis of the relationship between PA and AFT scores in the older adults with DM

	β (95% CI)	*P* - value
One - line linear regression model	0.09 (0.05, 0.13)	< 0.001
**Two - piecewise linear regression model**		
PA < 4.9 (100*MET-minutes/week)	0.45 (0.20, 0.70)	0.001
PA ≥ 4.9 (100*MET-minutes/week)	0.01 (− 0.07, 0.09)	0.864
Log-likelihood ratio test		< 0.001

Age, gender, race, marital status, education, PIR, alcohol drinkers, smokers, BMI, and other medical conditions were adjusted

**Table 4:** Threshold effect analysis of the relationship between PA and DSST scores in the older adults with DM

	β (95% CI)	*P* - value
One - line linear regression model	0.14 (− 0.06, 0.34)	0.164
**Two - piecewise linear regression model**		
PA < 11.2 (100*MET-minutes/week)	0.55 (0.20, 0.89)	0.004
PA ≥ 11.2 (100*MET-minutes/week)	− 0.15 (− 0.35, 0.05)	0.121
Log-likelihood ratio test		< 0.001

Age, gender, race, marital status, education, PIR, alcohol drinkers, smokers, BMI, and other medical conditions were adjusted

## Discussion

In this population-based study, we found a positive correlation between PA and cognitive function in older adults with DM. Furthermore, our analysis also revealed a nonlinear relationship between exercise and cognitive performance. Specifically, cognitive test scores improved substantially with increasing exercise volume but began to slow in enhancement after surpassing 490 MET-minutes/week for the AFT and 1,120 MET-minutes/week for the DSST. Our results suggested that PA had a threshold effect on cognitive function in older adults with DM, and these saturation points represented the optimal balance between exercise volume and cognitive benefits.

Our study indicated a positive association between PA volume and cognitive function in the older population with DM, which was consistent with the results of previous studies in older individuals [47, 48]. Data from the China Health and Retirement Longitudinal Study showed that older adults engaging in specific MET-level physical activities had better cognitive function than inactive individuals [49]. A research based on NHANES short-sleep older adults found that exercise volume was positively associated with cognitive function and had been linked to increased expression of brain chemicals [45]. While the exact mechanisms by which exercise improves cognitive function remain unclear, existing evidence suggests that exercise can provide neuroprotection through various pathways, such as promoting neuroplasticity, reducing inflammation, and enhancing cerebral blood flow, among others [25]. Studies have demonstrated that PA modulates the function and structure of specific brain regions, such as the hippocampus, which is critical for learning and memory [50, 51]. Exercise promotes neurogenesis, enhances synaptic plasticity, and increases dendritic spine density, particularly in the hippocampus and dentate gyrus [25, 52]. Additionally, PA can stimulate the production of neurotrophic factors, reduce neuroinflammation, and lower oxidative stress levels [51, 53, 54]. Furthermore, exercise has been shown to enhance glucose regulation and improve insulin sensitivity, thereby indirectly contributing to better cognitive outcomes in individuals with DM [31, 55, 56].

Through a dose-response investigation of the relationship between PA and cognitive function in older adults with DM, our study found a threshold effect of exercise. Optimal cognitive benefits were achieved at approximately 490 MET-min/week for AFT scores, equivalent to about 123 min of moderate-intensity activity per week, and at 1,120 MET-min/week for DSST scores, equivalent to about 280 min of moderate-intensity activity per week. Both thresholds fall within or slightly below the WHO 2020 recommendation of 150–300 min of moderate-intensity exercise per week for older adults, suggesting that current guidelines already encompass the cognitive-optimal dose [57].

The findings of our study showed a reverse U-shaped link between PA and cognitive function, consistent with prior research that indicated moderate-to-mild exercise was associated with better cognitive performance than vigorous exercise [58–61]. A randomized clinical trial also demonstrated a similar inverted U-shaped dose-response relationship, with moderate exercise volumes having a most clinically meaningful positive effect on cognitive outcomes [62]. Furthermore, a meta-analysis of 44 cohort studies highlighted a U-shaped dose-response relationship between exercise and cognition in older adults, showing that cognitive function does not always increase with increased PA dose, which aligns the findings presented here [63].

In addition, several potential mechanisms may account for this reverse U-shaped association [51, 64]. Recent research indicates that the brain maintains an independent glucose microsystem which protects neurons from transient hypoglycaemia [30, 65]. When this microsystem is perturbed, brain hyperglycaemia, glucotoxicity and insulin resistance emerge and disrupt insulin signalling, a primary driver of cognitive impairment. Meanwhile chronic hyperglycaemia, insulin resistance and low grade inflammation damage the cerebral microvasculature, reduce BDNF levels, and through oxidative stress and advanced glycation end products promote Aβ accumulation and tau hyper phosphorylation, thereby accelerating cognitive decline [66–68]. Moderate exercise lowers fasting glucose and HbA1c, up-regulates BDNF, improves insulin sensitivity and restores brain insulin signalling, directly counteracting these diabetes-specific insults [31, 56, 65, 69]. This could potentially explain why a relatively low threshold of exercise (e.g., 490 MET-min/week for AFT) was sufficient to yield significant cognitive benefits in our DM cohort. However, excessive exercise can lead to muscle damage, persistent fatigue, elicit pro-inflammatory responses and physiological stress that negate its potential benefits [70–72]. Given the common presence of comorbidities and age-related frailty in older adults with DM, exercise dose should be considered individually and controlled accordingly. Our identification of an optimal threshold therefore provides a pragmatic target for this special population, maximizing cognitive benefit while minimizing the risk of overexertion. This dose equates to 30 min of brisk walking at 3–4 mph (6 METs) on four days per week, or 20 min of running at 3.6 mph (8.5 METs) on three days per week, with intensity and duration adjusted for individual frailty and comorbidities.

The principal strength of the present study lies in its pioneering exploration of the dose-response relationship between PA and cognitive function in older adults with DM. This result was achieved using a large, nationally representative sample from the NHANES database. To enhance the reliability of our findings, we employed adjusted weighted regression analyses and three models to account for potential confounders. Additionally, we conducted stratified analyses to further validate the robustness of these results. Finally, our study demonstrates that the optimal PA dose should be matched to the predominant cognitive deficit: 490 MET-min/week (123 min of moderate-intensity activity) for executive/verbal decline (low AFT) and 1,120 MET-min/week (280 min of moderate-intensity activity) for processing-speed or attentional decline (low DSST). Thus, clinicians can match the exercise volume to the patient’s cognitive profile while remaining within the WHO recommended range of 150–300 min/week, providing a practical, individualised exercise prescription for older adults with DM.

Despite the strengths of this study, the results should be interpreted with caution due to the following limitations. First, the cross-sectional nature of the data limits our ability to infer causal or temporal relationships between PA and cognitive outcomes. Second, the self-reported measures of PA volume in NHANES questionnaires may introduce recall or reporting bias. Future studies incorporating objective and precise measurement methods, such as pedometers and accelerometers, could help validate this association. Third, although we comprehensively accounted for possible covariates in this study, other potential confounders, such as biological and genetic factors, could still influence our results. Furthermore, while our findings highlight the relationship between weekly PA volume and cognitive outcomes in older adults with DM, the specific impact of long-term exercise exclusively on cognitive enhancement remains uncertain. The cognitive benefits linked to sustained exercise regimens in this population warrant further exploration through longitudinal research.

## Conclusions

Drawing on data from the NHANES, our study revealed an inverted U-shaped association between PA and cognitive function in older adults with DM. Additionally, a dose-response analysis identified a threshold effect in this population, indicating that performing up to 490 MET-minutes/week of exercise was associated with improved cognitive performance. By integrating exercise thresholds into prevention programs for cognitive decline in older individuals with DM, we can maximize the benefits of exercise. However, the long-term effects of exercise on cognitive enhancement remain unclear. Future research should focus on clarifying the relationship between exercise duration, intensity, and cognitive function in this population, as well as investigating individualized exercise regimens to maximize cognitive health benefits.

## Supplementary Information

Below is the link to the electronic supplementary material.

Fig. S1. Weighted linear regression of stratified results for PA (as a continuous variable) with the score of the Animal Fluency test.

Fig. S2. Weighted linear regression of stratified results for PA (as a categorical variable) with the score of the Animal Fluency test. PA: Physical Activity. PA 1 = None (< 1 MET-min/week), PA 2 = Inadequate activity (1 to 600 MET-min/week), and PA 3 = Adequate activity (≥ 600 MET-min/week).

Fig. S3. Weighted linear regression of stratified results for PA (as a continuous variable) with the score of the Digit Symbol Substitution test.

Fig. S4. Weighted linear regression of stratified results for PA (as a categorical variable) with the score of the Digit Symbol Substitution test. PA: Physical Activity. PA 1 = None (< 1 MET-min/week), PA 2 = Inadequate activity (1 to 600 MET-min/week), and PA 3 = Adequate activity (≥ 600 MET-min/week).

## Data Availability

The publicly available datasets used in this study were obtained from the National Health and Nutrition Examination Survey (NHANES) website: http://www.cdc.gov/nchs/nhanes/.
